# Identification of Parasitic Infections by Analyzing Honeybees, Honey, and Pollen Using Droplet Digital RT-PCR

**DOI:** 10.3390/microorganisms13071487

**Published:** 2025-06-26

**Authors:** Luigi Jacopo D’Auria, Andrea Mancusi, Yolande Thérèse Rose Proroga, Irene Dini, Tiziana Cardellicchio, Orlandina Di Maro, Sabato De Vita, Marica Egidio, Raffaele Marrone, Giuseppe Rofrano

**Affiliations:** 1 Centro di Referenza Nazionale per L’analisi e lo Studio delle Correlazioni tra Ambiente, Animali e Uomo, Istituto Zooprofilattico Sperimentale del Mezzogiorno, 80055 Portici, Italy; jacopoluigi.dauria@izsmportici.it (L.J.D.); sabato.devita@izsmportici.it (S.D.V.); giuseppe.rofrano@izsmportici.it (G.R.); 2 Department of Food Security Coordination, Istituto Zooprofilattico Sperimentale del Mezzogiorno, 80055 Portici, Italy; yolande.proroga@izsmportici.it (Y.T.R.P.); orlandina.dimaro@izsmportici.it (O.D.M.); 3 Department of Pharmacy, University of Naples Federico II, 80138 Naples, Italy; 4 Department of Veterinary Medicine and Animal Production, University of Naples Federico II, 80138 Naples, Italy; marica.egidio@libero.it (M.E.); raffaele.marrone@unina.it (R.M.)

**Keywords:** bioindicators, droplet digital RT-PCR, hive products, honey, honeybees, pollen, protozoan parasites

## Abstract

*Toxoplasma gondii*, *Giardia intestinalis*, and *Cryptosporidium* spp. are common pathogens that contaminate water and food. They can pose serious health risks, especially to vulnerable groups like immunocompromised individuals, pregnant women, young children, and aging people. An all-encompassing approach to minimizing transmission involves identifying effective techniques for detecting, treating, and preventing protozoan parasites. This study confirmed the effectiveness of a Droplet Digital Reverse Transcription Polymerase Chain Reaction (dd RT-PCR) method for quickly and accurately identifying *Toxoplasma gondii*, *Giardia intestinalis*, and *Cryptosporidium* species in honeybees, honey, and pollen by using ISO 17468 and ISO 16140 standard guidelines. The study evaluated honeybee (*n* = 16), honey (*n* = 12), and pollen (*n* = 8) samples collected from various apiaries in Southern Italy between June and September 2023. The results showed that honeybees, honey, and pollen can be considered bioindicators of infections by *T. gondii*, *G. intestinalis*, and *Cryptosporidium* spp. Furthermore, pollen, along with honey to a lesser degree, can serve as significant indicators for evaluating food safety. Therefore, it is essential to monitor their quality and purity due to environmental influences.

## 1. Introduction

Soil contamination by protozoan parasites poses a significant infection risk to humans and animals [1]. *Toxoplasma gondii* can cause asymptomatic or mild symptomatic infections that are often resolved independently in individuals with a healthy immune system. However, it can cause central nervous system diseases, myocarditis, and pneumonitis in fetuses and immunosuppressed subjects. Latent infections may also be associated with neuropsychiatric disorders. During pregnancy, infection can cause congenital transmission, resulting in complications such as miscarriage, stillbirth, premature birth, neonatal mortality, and various symptoms in newborns [2]. *Giardia intestinalis* and *Cryptosporidium* spp. are responsible for significant intestinal disorders. For instance, human cryptosporidiosis primarily manifests as severe watery diarrhea, mild fever, abdominal discomfort, vomiting, nausea, and weight loss. While often asymptomatic or mild in healthy individuals, they can be severe and fatal in immunocompromised patients. Acute giardiasis symptoms resemble those of cryptosporidiosis [3]. All three of these protozoa are characterized by high resilience and survival, as they can persist for months in the environment. Their survival in the soil depends on soil chemistry, temperature, and humidity [4,5]. Contaminated soil with protozoan cysts (*G. intestinalis*) or oocysts (*T. gondii*, *Cryptosporidium* spp.) act as a pabulum matrix for contaminating water sources and agricultural products like fruits and vegetables [6]. Previous research has shown that oocyst survival is higher during the rainy season, as moisture facilitates their spread [7]. Additionally, sandy and loamy soils have the highest contamination rates, as they enhance the survival chances of these parasites [8]. The World Health Organization [1] promoted a “One Health approach” for managing zoonotic diseases [9]. It is a collaborative strategy that operates at various scales to achieve optimal health by recognizing the interconnections among humans, animals, plants, and the environment. Real-time environmental monitoring, using biotic or abiotic systems through physical, chemical, and genomic analyses, can be crucial for tracking changes and promoting human health. This study assessed the prevalence and parasitic load of *T. gondii*, *G. intestinalis*, and *Cryptosporidium* spp. DNA in honeybee, honey, and pollen samples from selected apiaries of southern Italy through a Digital RT-PCR technique. The European honeybee (*Apis mellifera*) is an ideal biomonitoring candidate due to its extensive foraging behavior, during which it inadvertently collects chemical and microbiological pollutants. As a result, the hive products, particularly honey, can indicate both contaminant exposure and potential food safety risks. Their widespread distribution and human management further enhance their potential in the context of environmental and food safety monitoring [10,11,12].

## 2. Materials and Methods

### 2.1. Study Area and Sampling

**Figure 1:**
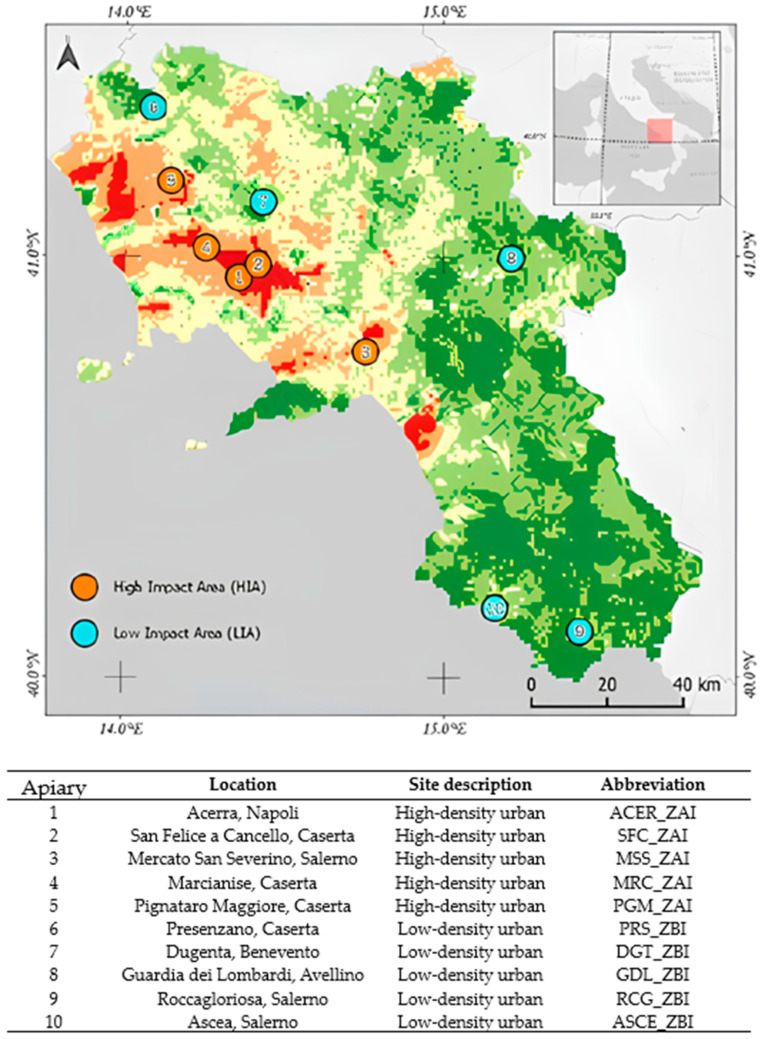
Map showing the sampling sites, classified as high-impact areas (HIAs) and low-impact areas (LIAs), investigated in this study in southern Italy.

This study was conducted in the Campania, a southern Italy region (Figure 1). It is characterized by a typical Mediterranean temperate climate and progressively continental features, particularly in the mainland and mountainous landscapes.

The apiaries were selected because of a risk index based on five environmental variables (i.e., pollution, land use, hydrographic network, air quality, and bee density) and classified as high-impact areas (HIAs) or low-impact areas (LIAs).

From June to September 2023, samples of honeybees (*n* = 16, S1–S16), honey (*n* = 12, S17–S28), and pollen (*n* = 8, S29–S36) were collected from 10 different apiaries (Table 1 and Figure 1) in collaboration with apiary owners that monitored the presence of *Varroa destructor* in the hive potentially related to the infections. Bees were captured alive and collected using special cages (formerly under-basket cages) to monitor mortality, allowing for the safe and effective collection of samples without harming the insects [13]. The honey was collected directly from the brood combs inside the beehives, ensuring the maximum freshness and integrity of the sample. The pollen was obtained by installing front traps on the hives, which captured the pollen grains collected by the bees during their foraging activities. All samples were stored at −20 °C in specific 500 µL glass jars and delivered to the Animal Health Department of the Experimental Zooprophylactic Institute of Southern Italy (Portici, Italy) for molecular analyses.

### 2.2. DNA Extraction

This study was conducted on honeybees (*n* = 16 comprising 50 honeybees), honey (*n* = 12 containing 1 mL of honey), and pollen (*n* = 8 each of 10 g).

Every sample underwent molecular analysis to assess the potential presence of parasites (*T. gondii*, *G. intestinalis*, and *Cryptosporidium* spp.). Three replicates were performed at each concentration level. Honeybee DNA was obtained by adding 40 mL of Tris-glycine buffer (TGBE; Merck, Darmstadt, Germany) to sub-samples in a Falcon tube, vertexing for 10 s, and shaking in an orbital shaker (DLAB Scientific, Beijing, China) for 1 h at 150 rpm at room temperature. Then, the Falcon tubes were centrifuged for 30 min at 10,000× *g* at 4 °C. The pellet was resuspended in 1 mL of PBS pH 7.3 and transferred to a 1.5 mL tube at −20 °C. Pollen and honey DNA were extracted from each sample by diluting 1 mL of honey or 10 g of pollen with TGBE (Merck, Darmstadt, Germany) at a ratio of 1:10. Subsequently, the prepared samples were vortexed for 10 s and blended in an orbital shaker (DLAB Scientific, Beijing, China) for 1 h at 150 rpm at room temperature. Then, 1 mL from each sample was transferred into a 1.5 mL tube and stored at −20 °C.

The extraction process was carried out using the EGene-UP system (bioMerieux, Marcy-l’Étoile, France), along with NucliSENS reagents (bioMerieux, Marcy-l’Étoile, France) and magnetic silica (bioMérieux, Marcy-l’Étoile, France). A sample lysis was conducted following the ISO 15216-2:2019 [14]. Two milliliters of lysis buffer were added to the 500 μL sample, mixed, and incubated at room temperature for 10 min. The resulting mixture was centrifuged (Eppendorf, Hamburg, Germany) at 1800× *g* for 2 min at 4 °C to collect any sample droplets that may have adhered to the walls of the tubes and their caps. Subsequently, 50 μL of magnetic silica (bioMerieux, Marcy-l’Étoile, France) was incorporated and kept for 10 min at room temperature. The subsequent steps followed the manufacturer’s guidelines, utilizing an INTEGRA 1250 μL 8-channel FW 4.02 pipette (INTEGRA Biosciences Corp, Hudson, NY, USA). The magnetic silica was resuspended in 100 μL of Nuclisens^®^ extraction buffer 3 (bioMerieux, Marcy-l’Étoile, France). The DNA was eluted from the silica (temperature: 60 °C, time: 5 min, speed: 1400 rpm) and stored at −20 °C until it was analyzed using dd-PCR.

### 2.3. Droplet Digital RT-PCR (dd RT-PCR) for Parasitic Load Detection

Droplet digital RT-PCR was performed on Bio-Rad’s QX200 system (Bio-rad, Hercules, CA, USA), following the manufacturer’s guidelines. The reaction mixture, with a total volume of 20 µL, was composed of ddPCR Supermix for probes (10 μL; Bio-Rad, Hercules, CA, USA); forward, reverse, and probe (Table 2); and DNA (35–50 ng), along with primers and probes and nuclease-free water as necessary to obtain the final volume. The PCR amplification was performed using a CFX96 Deep Well instrument (Bio-Rad, Hercules, CA, USA) with an initial 60 min step at 50 °C, followed by 10 min at 95 °C, and then 45 cycles of 15 s at 95 °C and 45 s at 60 °C, concluding with a final extension at 98 °C for 10 min. The QX200 Droplet Reader analyzed positive droplets following the Poisson distribution. QuantaSoft software (version 1.7) counts both PCR-positive and PCR-negative droplets, enabling the absolute quantification of DNA. The results were expressed as the number of genomic copies per 1 μL of the reaction (gc/1 μL).

### 2.4. Test Performance Assessment

Solutions of *T. gondii* MBC047, *G. intestinalis* MBC119-R (2 × 10^4^ genomic copies gc/μL; Vircell Microbiologist, Granada, Spain), and *Cryptosporidium* spp. MBC126-R (2 × 10^4^ gc/μL. Vircell Microbiologist, Granada, Spain) were used as reference strains. The strains were diluted in DNase/RNase-free water to obtain three concentrations: 100 gc/μL, 10 gc/μL, and 1 gc/μL. Validation samples were prepared by inoculating the reference strains (100 gc/μL, 10 gc/μL, and 1 gc/μL) or sterile water (negative control) into honeybees, pollen, and honey samples that tested negative for the examined parasites. The negativity for *Toxoplasma gondii* was assessed utilizing the qPCR protocol recommended by the National Reference Centre for Toxoplasmosis in Palermo, Italy. In contrast, *Giardia intestinalis* and *Cryptosporidium* spp. were detected using the real-time PCR protocol established by Haque et al. [16].

The test’s sensitivity was assessed by calculating the Limit of Detection at a 95% probability (LOD95), utilizing ten replicates for each concentration of parasites.

The detection limit for the dd-PCR assay was established as the final serial dilution measured in 95% of the replicates. The limit of quantification (LOQ) was established at the minimum concentration, demonstrating a coefficient of variation percentage (CV%) below the acceptance threshold of 25% for quantitative methods [18].

The test precision was evaluated by controlling the intraday and interday intra-laboratory repeatability. The intraday repeatability was assessed by performing ten analyses on each dilution level of the samples (100, 10, and 1 gc/mL) on the same day, while the interday repeatability was determined by repeating the same analyses on two different days. Different operators performed the same analyses to confirm the reliability of the ddPCR method and to obtain the intra-laboratory repeatability.

### 2.5. Statistical Analyses

For the validation test, statistical analysis was performed by calculating the intra-assay coefficient of variation (CV%) for each dilution level (100, 10, and 1 gc/mL) and overall, using the following formula. A coefficient of variation (CV%) lower than 10% indicates no significant intra-laboratory variation.

(1) $$\mathrm{CV}\%=\frac{\mathrm{Standard}\;\mathrm{deviation}}{\mathrm{the}\;\mathrm{mean}\;\mathrm{value}\;\mathrm{for}\;\mathrm{each}\;\mathrm{level}}\times 100$$

Moreover, Chi-square and Fisher’s exact tests were performed separately for each sample matrix and each pathogen using IBM SPSS Statistics (version 29, IBM Analytics, Armonk, NY, USA) to compare them. Odds ratios (ORs) and *p*-values were calculated, and significance was set at *p* < 0.05. Finally, prevalence (%) and corresponding 95% confidence intervals (IC95%) were calculated using the Wilson score method to avoid bias associated with small sample sizes.

## 3. Results

### 3.1. Performance Evaluation of dd-PCR Assay

The ddPCR technique enabled highly sensitive DNA amplification, detecting concentrations as low as 1 genome copy per microliter (gc/μL).

The detection limit confirmed the method’s sensitivity at 95% confidence (LOD95), which was 10 genome copies per microliter (gc/μL) for pollen samples and 1 gc/μL for bee and honey samples. These values were consistent across the different pathogens tested, namely *T. gondii*, *G. intestinalis*, and *Cryptosporidium* spp., indicating a stable performance of the method across diverse biological matrices.

According to the Bio-rad (Bio-rad, Hercules, CA, USA) manual, a minimum of 1 droplet is required as the essential quantitative parameter. In this work, as indicated by Srisutham et al. [19], sample classification as positive was based on detecting at least two positive droplets, a criterion that ensured high reliability in infection identification. All infected replicates were correctly detected as positive, while negative controls showed no detectable signal, confirming the high specificity of the technique.

During the reactions, the number of generated droplets ranged from 9245 to 14,681, with an average of 12,847 droplets per reaction, underscoring the robustness and reproducibility of the system. The analysis revealed a clear separation between positive and negative droplets, with a minimal presence of intermediate or ambiguous droplets, further supporting the high specificity and efficiency of ddPCR.

Regarding diagnostic performance, ddPCR showed a sensitivity of 97.6% (95% CI: 86.4–99.9) and a specificity of 100%. The overall coefficient of variation (CV%) among positive replicates was 20.2%, with CV values increasing as the target concentration decreased, as shown in Table 3.

At higher concentrations (100 gc/μL), CV values remained low across all matrices and pathogens, ranging from 0.41% to 6.74%. However, at 10 gc/μL, the CV% increased notably, particularly in pollen samples, where values reached up to 48.23% for *Cryptosporidium* spp. At the lowest concentration (1 gc/μL), CV values were markedly higher, especially for *Giardia intestinalis* in honey (65%) and honeybee samples (51.22%), and for *Toxoplasma gondii* in the same matrices (49.33% and 37.89%, respectively). At this concentration, all targets in pollen were below the detection limit. These results emphasize the high precision of ddPCR at moderate-to-high DNA concentrations while highlighting the expected variability at the lower detection limits.

### 3.2. Honeybees, Honey, and Pollen Analyses

The screening of honeybees collected between June and September 2023 from different apiaries in the Campania region revealed the presence of seven samples positive for the *Toxoplasma* gene (prevalence: 43.8%; IC95%: 23.1–66.8), twelve for the *Giardia* gene (prevalence: 75%; IC95%: 50.5–89.8), and eight (prevalence: 50%; IC95%: 28–72) for the *Cryptosporidium* spp. gene (Table 4). No statistically significant differences (*p* > 0.05) related to seasonality were observed for any of the three pathogens.

Regarding the geographic distribution, the *Toxoplasma* gene was detected in Naples, Caserta, and Benevento; instead, the *Giardia* and *Cryptosporidium* spp. genes were in all five provinces of the Campania region.

The analysis of honey gathered from various apiaries in the Campania region between June and September 2023 indicated that three samples were positive for the *Toxoplasma gondii* gene (prevalence: 25%; IC95%: 8.9–53.2), one for the *Giardia intestinalis* gene (prevalence: 8.3%; IC95%: 1.5–35.4), and one for the *Cryptosporidium* spp. gene (prevalence: 8.3%; IC95%: 1.5–35.4). No statistically significant differences (*p* > 0.05) related to seasonality were observed for any of the three pathogens (Table 5).

Regarding the geographic distribution, the *Toxoplasma gondii* gene was detected in Caserta and Salerno, the Giardia intestinalis in Caserta, and *Cryptosporidium* spp. in Benevento province.

The examination of pollen collected from different apiaries in the Campania region from June to September 2023 revealed that five samples tested positive for the *Toxoplasma gondii* gene (prevalence: 62.5%; IC95%: 30.6–86.3), three for the *Giardia intestinalis* gene (prevalence: 37.5%; IC95%: 13.7–69.4), and four for the *Cryptosporidium* spp. gene (prevalence: 50%; IC95%: 21.5–78.5). No statistically significant differences (*p* > 0.05) related to seasonality were observed for any of the three pathogens.

Regarding the geographic distribution, the *Giardia intestinalis* gene was detected in Avellino, Salerno, and Benevento; instead, the *Toxoplasma gondii* and *Cryptosporidium* spp. genes were in all five provinces of the Campania region (Table 6).

## 4. Discussion

*T. gondii*, *G. intestinalis*, and *Cryptosporidium* spp. are prevalent zoonotic foodborne protozoa; however, systematic food safety controls for these parasites are not applied, allowing food safety concerns to persist. This deficiency is primarily attributed to insufficient specific regulations and standardized detection methods for identifying parasites in food matrices [18]. There is an urgent need for rapid and standardized detection methods to address the growing threat of protozoan parasites in our food supply. Advances in molecular biology and diagnostic technologies offer promising avenues for accurately and timely identifying these pathogens. Examining the parasitic load and prevalence in various food products is vital for understanding the level of contamination and developing targeted interventions. Providing occurrence data pertinent to food safety risk managers is essential, clarifying the implications of negative, positive, and quantifiable samples. This study assessed the efficacy of ddPCR technology in identifying target parasites. ddPCR, which eliminates the need for sequencing, utilizes fluorescent-labeled, target-specific probes for identification, enabling the rapid and sensitive detection of low levels of parasite DNA [20]. The primary advantage of ddPCR is its utilization of water–oil droplet emulsion technology, which divides samples into approximately 20,000 droplets, thereby enhancing sensitivity, accuracy, and precision in detecting low target quantities. This method eliminates the need for a standard curve in DNA quantification, allowing for absolute counting through Poisson statistics and enabling the detection of subtle differences in target DNA copy numbers. ddPCR also offers superior reproducibility to qPCR, as it is less affected by operator and environmental variables, reducing errors from pipetting and standard curve preparation. Additionally, ddPCR is less sensitive to PCR inhibitors, leading to greater consistency among replicates and improved repeatability [15].

In the absence of established performance criteria for detecting parasites in food, the test performance was evaluated using the guidelines from ISO 17468 [21], ISO 16140-1 [22], and ISO 16140-2 [23], which were initially formulated for bacterial detection [24].

ISOs are typically employed with slight adjustments to optimize parasite identification, ensuring accuracy and adaptability across different samples. Qualitative methods require LOD and sensitivity, while quantitative methods focus on LOQ and percentage recovery, both needing artificial contamination studies and interlaboratory trials. Inclusivity and exclusivity do not require artificial contamination. ISO 16140–2:2016 suggests evaluating sensitivity first; however, for parasites, determining the Limit of Detection (LOD) initially is more logical. The recommended number of food items and biological replicates can be challenging for non-culture methods due to the limited availability of parasites and the difficulties associated with processing large sample volumes [24].

The ddPCR assay demonstrated excellent diagnostic performance, with a specificity of 100% and a sensitivity of 97.6% (95% CI), across all tested matrices and target pathogens (*T. gondii*, *G. intestinalis*, and *Cryptosporidium* spp.). These results support the method’s potential as a reliable tool for standardized food safety inspections, which can reduce the public health risks associated with these parasitic contaminants.

This study evaluated the presence of *T. gondii*, *G. intestinalis*, and *Cryptosporidium* spp. in the five provinces of the Campania region. The study areas were chosen to address key environmental concerns, including the presence of surface water bodies, intensive agricultural activities, and significant human impact. This selection aimed to facilitate precise environmental and parasitological monitoring using bioindicators, ensuring an insightful analysis of ecological conditions and potential risks [25].

The data show that the parasite’s presence in Campania varied across the provinces. Notably, the honeybee samples with a high rate of parasite (*T. gondii*, *G. intestinalis*, and *Cryptosporidium* spp.) gene contamination were those that came from Palma Campania, followed by Guardia Lombardi, Dugenta, Acerra, and Presenzano. The geographical location, climate, and agronomic practices all affected parasitosis levels in the different countries. In the two weeks leading up to the sample collection, Palma Campania, Guardia Lombardi, and Acerra experienced rainfall and higher humidity levels (Figure S1). This finding is consistent with the conclusions of Berrouch et al. [26], who identified humidity and precipitation as key factors in the spread of parasites. The meteorological conditions cannot support the data collected in the provinces of Acerra, Dugenta, and Presenzano before the sample collection (Figure S2).

It is worth mentioning that the Acerra and Dugenta areas have a strong agricultural vocation. The Dugenta province is famous for its wines, which are marked by the “Dugenta” geographical indication. Many farmers use irrigation in the warm months (June and August) to boost agricultural production yields. The water used for irrigation, combined with rising temperatures, may affect local humidity levels.

In Presenzano, a closed-cycle hydroelectric facility with two reservoirs holding about 6 million cubic meters can also influence humidity during hot periods.

The data on the hive products revealed the presence of *T. gondii*, *G. intestinalis*, and *Cryptosporidium* spp. in both pollen and honey samples, albeit with lower detection frequencies than those observed in honeybees. This finding suggests that, although all matrices are exposed to similar environmental sources, they exhibit different capacities for accumulating or preserving protozoan pathogens, also depending on the localization of the raw material from which they originate (e.g., external or internal parts of flowers) and on how honeybees process them [27].

Notably, pollen exhibited a higher prevalence compared to honey samples (13.9% and 50%, respectively), with no statistically significant differences (*p* > 0.05), likely due to its direct collection from the external parts of flowers, which may be more contaminated by environmental particles, such as rainwater, organic fertilizers, or dust containing oocysts, and less transformation by bees [6,7,8,27].

In contrast, honey showed a lower contamination incidence than the other two sample types. This is likely due to its raw material origin (the nectar is located within floral structures inherently less exposed to environmental contaminants than the external parts), low water activity, high sugar content, acidic pH, and the presence of compounds such as polyphenols, 1,2-dicarbonyl compounds, bee defensin-1, and substances such as hydrogen peroxide, which may reduce pathogen viability [28,29], and to the biofilter role of honeybees, which mitigate contamination in nectar during the production process of honey, even under conditions of high environmental pollution [27].

Nevertheless, the detection of protozoan DNA, albeit limited, confirms that honey is not entirely exempt from environmental contamination. Our data are in accordance with those reported by Flamminii, Salkova, and González-Alcaraz [27,30,31], who also identified pollen as a more effective bioindicator of harmful pollutants such as heavy metals, radionuclides, and pathogenic microorganisms, compared to honey for the reasons outlined above.

From a geographical perspective, the heterogeneous distribution of *T. gondii*, *G. intestinalis*, and *Cryptosporidium* spp. genes across the three different matrices (honeybees, honey, and pollen) and the five provinces of the Campania region is likely the result of a complex interplay between agronomic practices, climatic conditions, and environmental contamination sources. Agricultural intensity exhibits substantial inter-provincial variation, with some areas characterized by intensive crop cultivation or high-density livestock farming. These activities can increase the environmental loads of protozoan (oo)cysts through the use of organic fertilizers, runoff, or irrigation with untreated water. Climatic differences, such as higher precipitation and humidity, can further facilitate the dissemination of protozoan contaminants in the environment [27]. Thus, detecting protozoan DNA in different provinces depending on the matrix likely reflects localized environmental conditions and matrix-specific susceptibility to contamination.

Finally, the temporal analysis revealed that July recorded the highest prevalence (June: 50%; July: 80%; August: 66.6%; September: 25%) of contaminated honey and pollen samples, aligning with the honeybees’ findings. This peak may be attributed to increased foraging activity during the summer months and/or a higher environmental pathogen load during this period, supported by climatic conditions favorable to protozoan persistence [4].

## 5. Conclusions

The research conducted following the ISO 17468 and ISO 16140 standards confirmed the effectiveness of the ddRT-PCR method for the rapid and precise identification of *Toxoplasma gondii*, *Giardia intestinalis*, and *Cryptosporidium* genes in honeybees, honey, and pollen. The findings indicate that honeybees, along with pollen and honey, could potentially act as vectors for the transmission of *T. gondii*, *G. intestinalis*, and *Cryptosporidium* spp. Honey and pollen, with their direct use as a food product, can serve as both a valuable indicator of environmental contamination and a reliable marker of food safety. Further studies involving a larger and standardized number of honey, pollen, and honeybee samples are currently underway to confirm these preliminary results.

## Figures and Tables

**Table 1:** Samples (i.e., honeybees, honey, and pollen) and collection sites in the Campania region.

Sample		Sampling Period	Sampling Site
Honey bees	S1	June	Nocera (SA)
	S2	June	Guardia Lombardi
	S3	June	Palma Campania (NA)
	S4	July	Presenzano (CE)
	S5	July	Acerra (NA)
	S6	July	Pignataro (CE)
	S7	July	Marcianise (CE)
	S8	July	Mercato S.S. (SA)
	S9	July	Nocera (SA)
	S10	July	Palma Campania (NA)
	S11	July	Mercato S.S. (SA)
	S12	August	San Felice Cancello (CE)
	S13	August	San Felice Cancello (CE)
	S14	August	Dugenta (BN)
	S15	September	Roccagloriosa (SA)
	S16	September	Ascea (SA)
Honey	S17	June	Guardia Lombardi (AV)
	S18	June	Guardia Lombardi (AV)
	S19	July	Acerra (NA)
	S20	July	Pignataro (CE)
	S21	July	Marcianise (CE)
	S22	July	Presenzano (CE)
	S23	July	Mercato S.S. (SA)
	S24	August	Dugenta (BN)
	S25	August	Dugenta (BN)
	S26	August	San Felice Cancello (CE)
	S27	September	Presenzano (CE)
	S28	September	Ascea (SA)
Pollen	S29	June	Guardia Lombardi (AV)
	S30	June	Guardia Lombardi (AV)
	S31	July	Acerra (NA)
	S32	July	Acerra (NA)
	S33	July	Mercato S.S. (SA)
	S34	August	Dugenta (BN)
	S35	August	Dugenta (BN)
	S36	August	San Felice Cancello (CE)

**Table 2:** Primers and probes used for the parasites’ detection.

Primer Name	Sequence 5′–3′	Concentrations	Reference
AF1	CACAGAAGGGACAGAAGT	500 nM	[15]
AF2	TCGCCTTCATCTACAGTC	500 nM	
AF probe	FAM—CTCTCCTCCAAGACGGCTGG—BHQ	250 nM	
GD 80 For	GACGGCTCAGGACAACGGTT	360 nM	[16]
GD 127 Rev	TTGCCAGCGGTGTCCG	360 nM	
GD 105 probe	FAM—CCCGCGGCGGTCCCTGCTAG—BHQ1	90 nM	
COWP For	CAAATTGATACCGTTTGTCCTTCTG	600 nM	[17]
COWP Rev	GGCATGTCGATTCTAATTCAGCT	600 nM	
Crypto probe	FAM—TGCCATACATTGTTGTCCTGACAAATTGAAT—BHQ1	240 nM	

BHQ: Black Hole Quencher.

**Table 3:** Honey, pollen, and honeybees (CV%) for each concentration level of the parasite sample.

		100 gc/μL	10 gc/μL	1 gc/μL
Honeybee	*Toxoplasma gondii*	0.62	2.93	37.89
	*Giardia intestinalis*	0.52	4.92	51.22
	*Cryptosporidium* spp.	0.41	5.72	51.07
Honey	*Toxoplasma gondii*	0.64	7.87	49.33
	*Giardia intestinalis*	0.64	7.86	65
	*Cryptosporidium* spp.	0.83	11.67	50
Pollen	*Toxoplasma gondii*	3.75	29.88	NOT DETECTED
	*Giardia intestinalis*	6.74	43.09	NOT DETECTED
	*Cryptosporidium* spp.	3.84	48.23	NOT DETECTED

**Table 4:** Parasite-positive honeybee samples.

Sampling Period	Sample	Sampling Site	*Toxoplasma gondii*		*Giardia intestinalis*		*Cryptosporidium* spp.	
			DDPCR	copies/μL	DDPCR	copies/μL	DDPCR	copies/μL
June	S1	Nocera (SA)	−	−	+	4.6	−	−
June	S2	Guardia Lombardi (AV)	+	14.1	+	13.8	+	12.4
June	S3	Palma Campania (NA)	+	21.5	+	18.8	+	6.8
July	S4	Presenzano (CE)	+	2.5	+	1.1	+	1
July	S5	Acerra (NA)	+	3.2	+	5.6	+	4.6
July	S6	Pignataro (CE)	+	2.1	+	4.3	−	−
July	S7	Marcianise (CE)	−	−	−	-	−	−
July	S8	Mercato S.S. (SA)	−	−	+	1.6	−	−
July	S9	Nocera (SA)	−	−	+	9.6	+	19.2
July	S10	Palma Campania (NA)	−	−	+	6	+	5.9
July	S11	Mercato S.S. (SA)	−	−	−	-	−	−
August	S12	San Felice Cancello (CE)	+	1	+	1.6	+	0.7
August	S13	San Felice Cancello (CE)	−	−	−	-	−	−
August	S14	Dugenta (BN)	+	3.7	+	6.5	+	4.3
September	S15	Roccagloriosa (SA)	−	−	−	-	−	−
September	S16	Ascea (SA)	−	−	+	2.3	−	−
Prevalence (%)			43.75		75		50	
IC 95%			23.1–66.8		50.5–89.8		28–72	

+ = positive samples; − negative samples.

**Table 5:** Parasite-positive honey samples.

Sampling Period	Sample	Sampling Site	*Toxoplasma gondii*		*Giardia intestinalis*		*Cryptosporidium* spp.	
			DDPCR	copies/μL	DDPCR	copies/μL	DDPCR	copies/μL
June	S17	Guardia Lombardi (AV)	−	−	−	−	−	−
June	S18	Guardia Lombardi (AV)	−	−	−	−	−	−
July	S19	Acerra (NA)	−	−	−	−	−	−
July	S20	Pignataro (CE)	+	1.5	−	−	−	−
July	S21	Marcianise (CE)	−	−	+	0.13	−	−
July	S22	Presenzano (CE)	−	−	−	−	−	−
July	S26	Mercato S.S. (SA)	+	1.2	−	−	−	−
August	S24	Dugenta (BN)	−	−	−	−	−	−
August	S23	Dugenta (BN)	−	−	−	−	+	0.13
August	S25	San Felice Cancello (CE)	−	−	−	−	−	−
September	S27	Presenzano (CE)	−	−	−	−	−	−
September	S28	Ascea (SA)	+	0.8	−	−	−	−
Prevalence (%)			25		8.3		8.3	
IC 95%			8.9–53.2		1.5–35.4		1.5–35.4	

+ = positive samples; − negative samples.

**Table 6:** Parasite-positive pollen samples.

Sampling Period	Sample	Sampling Site	*Toxoplasma gondii*		*Giardia intestinalis*		*Cryptosporidium* spp.	
			DDPCR	copies/μL	DDPCR	copies/μL	DDPCR	copies/μL
June	S29	Guardia Lombardi (AV)	+	7.8	+	7.8	+	7.9
June	S30	Guardia Lombardi (AV)	−	−	−	−	−	−
July	S31	Acerra (NA)	+	1.1	−	−	−	−
July	S32	Acerra (NA)	−	−	−	−	−	−
July	S33	Mercato S.S. (SA)	+	24.3	+	14.9	+	30.6
August	S34	Dugenta (BN)	+	0.7	+	0.4	+	1.2
August	S35	Dugenta (BN)	−	−	−	−	−	−
August	S36	San Felice Cancello (CE)	+	2.2	−	−	+	4.4
Prevalence (%)			62.5		37.5		50	
IC 95%			30.6–86.3		13.7–69.4		21.5–78.5	

+ = positive samples; − negative samples.

## Data Availability

The original contributions presented in this study are included in the article/Supplementary Material. Further inquiries can be directed to the corresponding authors.

## Supplementary Materials

The following supporting information can be downloaded at: https://www.mdpi.com/article/10.3390/microorganisms13071487/s1, Figure S1: Climatic conditions in the two weeks before sample collection in the Palma Campania and Guardia Lombardi provinces with high parasite prevalence. Figure S2: Climatic conditions in the two weeks before sample collection in the Acerra, Dugenta, and Presenzano provinces with high parasite prevalence.
