# Supplementary material for: Identification of Parasitic Infections by Analyzing Honeybees, Honey, and Pollen Using Droplet Digital RT-PCR
Source: Microorganisms. 2025 Jun 26;13(7):1487. doi: 10.3390/microorganisms13071487 (PMC12299701; doi:10.3390/microorganisms13071487)
Supplement: Supplementary file 1 [file microorganisms-13-01487-s001.zip › microorganisms-3693926-supplementary.pdf]

Palma Campania

1-06-2023	2-06-2023	3-06-2023	4-06-2023	5-06-2023
2.3 mm<br>16°C 27°C	2.8 mm<br>15°C 26°C	7 mm<br>16°C 27°C	15°C 27°C	1 mm<br>18°C 25°C
**6-06-2023**	**7-06-2023**	**8-06-2023**	**9-06-2023**	**10-06-2023**
17°C 25°C	17°C 28°C	17°C 28°C	17°C 28°C	19°C 26°C
**11-06-2023**	**12-06-2023**	**13-06-2023**	**14-06-2023**	**15-06-2023**
10.6 mm<br>18°C 25°C	10.4 mm<br>17°C 29°C	17°C 26°C	13.8 mm<br>18°C 27°C	3 mm<br>18°C 24°C

3B meteo -historical weather forecast Palma Campania available online https://www.3bmeteo.com/meteo/palma+campania/storico/202306

Guardia Lombardi

9-06-2023	10-06-2023	11-06-2023	12-06-2023	13-06-2023
13°C 22°C	13°C 19°C	12°C 19°C	11°C 19°C	13°C 20°C
**14-06-2023**	**15-06-2023**	**16-06-2023**	**17-06-2023**	**18-06-2023**
10.5 mm<br>11°C 20°C	20 mm<br>13°C 15°C	13°C 19°C	12°C 19°C	11°C 21°C
**19-06-2023**	**20-06-2023**	**21-06-2023**	**22-06-2023**	**23-06-2023**
12°C 24°C	14°C 26°C	16°C 28°C	19°C 31°C	21°C 29°C

3B meteo -historical weather forecast Guardia Lombardi available online https://www.3bmeteo.com/meteo/guardia+lombardi/storico/202306

Figure S1. Climatic conditions in the two weeks before sample collection in the Palma Campania and Guardia Lombardi provinces with high parasite prevalence.

Acerra

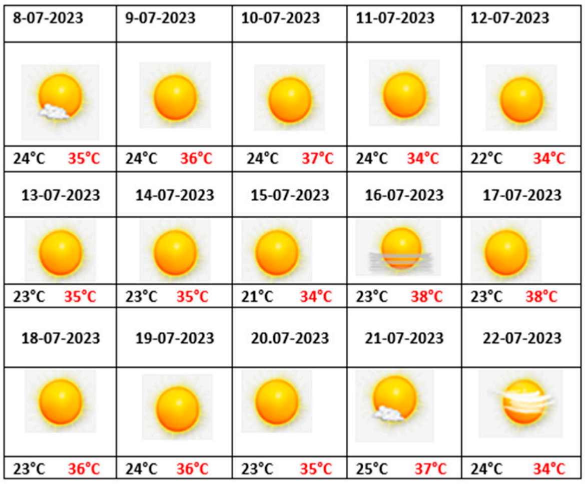

8-07-2023	9-07-2023	10-07-2023	11-07-2023	12-07-2023
24°C 35°C	24°C 36°C	24°C 37°C	24°C 34°C	22°C 34°C
13-07-2023	14-07-2023	15-07-2023	16-07-2023	17-07-2023
23°C 35°C	23°C 35°C	21°C 34°C	23°C 38°C	23°C 38°C
18-07-2023	19-07-2023	20.07-2023	21-07-2023	22-07-2023
23°C 36°C	24°C 36°C	23°C 35°C	25°C 37°C	24°C 34°C

3B meteo -historical weather forecast Acerra available online https://www.3bmeteo.com/meteo/acerra/storico

Dugenta

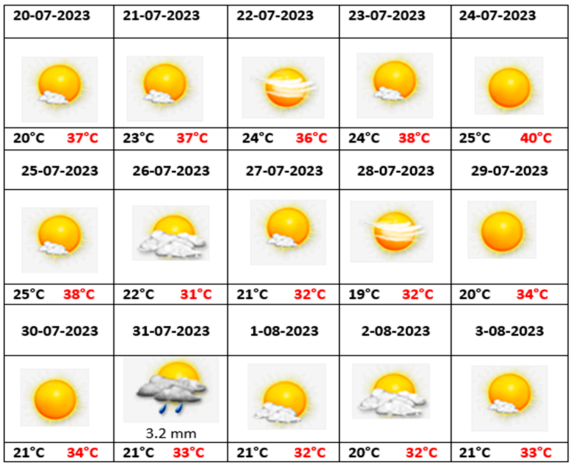

20-07-2023	21-07-2023	22-07-2023	23-07-2023	24-07-2023
20°C 37°C	23°C 37°C	24°C 36°C	24°C 38°C	25°C 40°C
25-07-2023	26-07-2023	27-07-2023	28-07-2023	29-07-2023
25°C 38°C	22°C 31°C	21°C 32°C	19°C 32°C	20°C 34°C
30-07-2023	31-07-2023	1-08-2023	2-08-2023	3-08-2023
	3.2 mm			
21°C 34°C	21°C 33°C	21°C 32°C	20°C 32°C	21°C 33°C

3B meteo -historical weather forecast Dugenta available online https://www.3bmeteo.com/meteo/dugenta/storico/202307

Presenzano

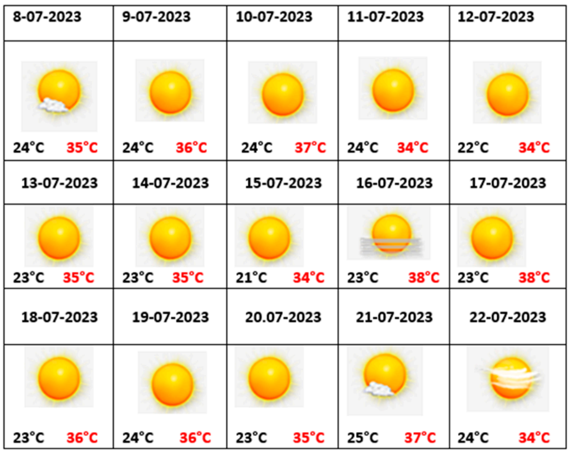

8-07-2023	9-07-2023	10-07-2023	11-07-2023	12-07-2023
24°C 35°C	24°C 36°C	24°C 37°C	24°C 34°C	22°C 34°C
13-07-2023	14-07-2023	15-07-2023	16-07-2023	17-07-2023
23°C 35°C	23°C 35°C	21°C 34°C	23°C 38°C	23°C 38°C
18-07-2023	19-07-2023	20.07-2023	21-07-2023	22-07-2023
23°C 36°C	24°C 36°C	23°C 35°C	25°C 37°C	24°C 34°C

3B meteo -historical weather forecast Presenzano available online https://www.3bmeteo.com/meteo/presenzano/storico/202307

Figure S2. Climatic conditions in the two weeks before sample collection in the Acerra, Dugenta, and Presenzano provinces with high parasite prevalence.
